# Associations of toothbrushing behavior with risks of vascular and non-vascular diseases in Chinese adults

**DOI:** 10.1111/eci.13634

**Published:** 2021-06-21

**Authors:** Zhenhuang Zhuang, Meng Gao, Jun Lv, Canqing Yu, Yu Guo, Zheng Bian, Ling Yang, Huaidong Du, Yiping Chen, Feng Ning, Huilin Liu, Junshi Chen, Zhengming Chen, Tao Huang, Liming Li, for the China Kadoorie Biobank (CKB) Collaborative Group, Members of the China Kadoorie Biobank collaborative group, International Steering Committee, Junshi Chen, Zhengming Chen, Robert Clarke, Rory Collins, Yu Guo, Liming Li, Jun Lv, Richard Peto, Robin Walters, International Co-ordinating Centre, Oxford, Daniel Avery, Ruth Boxall, Derrick Bennett, Yumei Chang, Yiping Chen, Zhengming Chen, Robert Clarke, Huaidong Du, Simon Gilbert, Alex Hacker, Mike Hill, Michael Holmes, Andri Iona, Christiana Kartsonaki, Rene Kerosi, Ling Kong, Om Kurmi, Garry Lancaster, Sarah Lewington, Kuang Lin, John McDonnell, Iona Millwood, Qunhua Nie, Jayakrishnan Radhakrishnan, Paul Ryder, Sam Sansome, Dan Schmidt, Paul Sherliker, Rajani Sohoni, Becky Stevens, Iain Turnbull, Robin Walters, Jenny Wang, Lin Wang, Neil Wright, Ling Yang, Xiaoming Yang, National Co-ordinating Centre, Beijing, Zheng Bian, Yu Guo, Xiao Han, Can Hou, Jun Lv, Pei Pei, Chao Liu, Yunlong Tan, Canqing Yu, 10 Regional Co-ordinating Centres: Qingdao CDC, Zengchang Pang, Ruqin Gao, Shanpeng Li, Shaojie Wang, Yongmei Liu, Ranran Du, Yajing Zang, Liang Cheng, Xiaocao Tian, Hua Zhang, Yaoming Zhai, Feng Ning, Xiaohui Sun, Feifei Li, Licang CDC, Silu Lv, Junzheng Wang, Wei Hou, Heilongjiang Provincial CDC, Mingyuan Zeng, Ge Jiang, Xue Zhou, Nangang CDC, Liqiu Yang, Hui He, Bo Yu, Yanjie Li, Qinai Xu, Quan Kang, Ziyan Guo, Hainan Provincial CDC, Dan Wang, Ximin Hu, Jinyan Chen, Yan Fu, Zhenwang Fu, Xiaohuan Wang, Meilan CDC, Min Weng, Zhendong Guo, Shukuan Wu, Yilei Li, Huimei Li, Zhifang Fu, Jiangsu Provincial CDC, Ming Wu, Yonglin Zhou, Jinyi Zhou, Ran Tao, Jie Yang, Jian Su, Suzhou CDC, Fang liu, Jun Zhang, Yihe Hu, Yan Lu, Liangcai Ma, Aiyu Tang, Shuo Zhang, Jianrong Jin, Jingchao Liu, Guangxi Provincial CDC, Zhenzhu Tang, Naying Chen, Ying Huang, Liuzhou CDC, Mingqiang Li, Jinhuai Meng, Rong Pan, Qilian Jiang, Jian Lan, Yun Liu, Liuping Wei, Liyuan Zhou, Ningyu Chen Ping Wang, Fanwen Meng, Yulu Qin, Sisi Wang, Sichuan Provincial CDC, Xianping Wu, Ningmei Zhang, Xiaofang Chen, Weiwei Zhou, Pengzhou CDC, Guojin Luo, Jianguo Li, Xiaofang Chen, Xunfu Zhong, Jiaqiu Liu, Qiang Sun, Gansu Provincial CDC, Pengfei Ge, Xiaolan Ren, Caixia Dong, Maiji CDC, Hui Zhang, Enke Mao, Xiaoping Wang, Tao Wang, Xi zhang, Henan Provincial CDC, Ding Zhang, Gang Zhou, Shixian Feng, Liang Chang, Lei Fan, Huixian CDC, Yulian Gao, Tianyou He, Huarong Sun, Pan He, Chen Hu, Xukui Zhang, Huifang Wu, Pan He, Zhejiang Provincial CDC, Min Yu, Ruying Hu, Hao Wang, Tongxiang CDC, Yijian Qian, Chunmei Wang, Kaixu Xie, Lingli Chen, Yidan Zhang, Dongxia Pan, Qijun Gu, Hunan Provincial CDC, Yuelong Huang, Biyun Chen, Li Yin, Huilin Liu, Zhongxi Fu, Qiaohua Xu, Liuyang CDC, Xin Xu, Hao Zhang, Huajun Long, Xianzhi Li, Libo Zhang, Zhe Qiu

**Affiliations:** 1 Department of Epidemiology and Biostatistics, School of Public Health, Peking University Health Science Center, Beijing, China; 2 Key Laboratory of Molecular Cardiovascular Sciences (Peking University), Ministry of Education, Beijing, China; 3 Peking University Institute of Environmental Medicine, Beijing, China; 4 Chinese Academy of Medical Sciences, Beijing, China; 5 Medical Research Council Population Health Research Unit at the University of Oxford, Oxford, United Kingdom; 6 Clinical Trial Service Unit & Epidemiological Studies Unit (CTSU), Nuffield Department of Population Health, University of Oxford, United Kingdom; 7 NCDs Prevention and Control Department, Qingdao CDC, Qingdao, China; 8 NCDs Prevention and Control Department, Hunan CDC, Hunan, China; 9 China National Center for Food Safety Risk Assessment, Beijing, China

**Keywords:** Dental public health, Epidemiology, Oral hygiene, Cardiovascular disease, Cohort study

## Abstract

Accumulating evidence has shown that poor oral hygiene is associated with increased risk of cardiometabolic diseases in Western populations. However, its relevance about the relationships in Chinese adults remains unclear. The China Kadoorie Biobank enrolled 512,715 adults aged 30-79 years in China during 2004-2008. Cox regression was used to estimate adjusted hazard ratios (HRs) for each disease associated with measures of oral hygiene. Overall 9.3% of the participants reported rarely or never brushing teeth at baseline. Participants who rarely or never brushed teeth had adjusted HR of 1.12 (95% CI: 1.09, 1.15) for MVE, with similar HRs for stroke (1.08, 1.05-1.12), intracerebral haemorrhage (1.18, 1.11-1.26), and pulmonary heart disease (1.22, 1.13-1.32) compared with those who brushed teeth regularly. Those who did not brush teeth also had increased risk of cancer (1.09, 1.04-1.14), chronic obstructive pulmonary disease (COPD) (1.12, 1.05-1.20), liver cirrhosis (1.25, 1.09-1.44) and all cause death (1.25, 1.21-1.28) but not type 2 diabetes (0.94, 0.86-1.03) and chronic kidney disease (0.98, 0.81-1.18). Among Chinese adults, we found that poor oral hygiene is associated with higher risks of major vascular disease, cancer, COPD, liver cirrhosis, and all cause deaths, but not type 2 diabetes and chronic kidney disease.

## Introduction

Globally, cardiovascular disease (CVD) accounted for more than 17 million deaths per year.^1,2^ In China, CVD remains the leading cause of death.^3^ Although a substantial proportion of CVD is attributable to traditional risk factors, other factors may also play a role in disease aetiology.^4^ Over the past few decades, there has been emerging evidence showing that oral hygiene may be associated with CVD risk.^5-9^

Good oral hygiene, in which toothbrushing has a central role, is important to prevent gingivitis and tooth decay,^10^ and reduce dental plaque formation.^11^ The Scottish Health Survey^8^ and a large-scale, cross-sectional Japanese study^9^ reported that self-reported poor oral hygiene is associated with higher risk of cardiometabolic diseases,^8,9^ and moderate systemic inflammatory response.^10^ However, the strength of the associations may differ substantially between different populations or within specific populations.^8,9^ It is worth noting that conflicting findings suggested the current evidence was insufficient to establish a causal association of oral hygiene with vascular disease.^12-15^ In addition, oral hygiene has been associated with non-vascular disease such as diabetes,^16^ chronic kidney disease,^17^ cancer,^18,19^ liver cirrhosis,^20^ and chronic obstructive pulmonary disease.^21^ Nevertheless, little evidence has suggested the extent of association between toothbrushing practices and vascular and non-vascular diseases among Chinese adults. Considering toothbrushing is one of the most familiar and easiest methods of improving oral hygiene, elucidating such association is of major public significance.

Therefore, in the present study, we investigated the associations between self-reported measure of toothbrushing behavior (as a proxy of oral hygiene) and the risk of cardiovascular events in a sample of adults from the China Kadoorie Biobank (CKB). In addition, we examined the association of toothbrushing behavior with non-vascular diseases such as diabetes, cancer, liver diseases and chronic kidney disease.

## Methods

### Study population

Detailed information on the study design, characteristics of the study participants, and survey methods in CKB have been reported elsewhere.^22,23^ During 2004-2008, 512,715 men and women aged 30-79 years were recruited from the general population in five rural and five urban areas in China. Participants were identified through public registry records and invited to attend study assessment clinics and about one-third of residents responded. The study was approved by the ethical review committee of the Chinese Center for Disease Control and Prevention and the Oxford Tropical Research Ethics Committee, University of Oxford. All participants provided written informed consent forms.

### Data collection

Information on covariate at baseline including region, sociodemographic factors (age, sex, marital status, highest education, occupation, household income), tobacco smoking, passive smoking, alcohol intake, fruit intake, vegetable intake, meat intake, physical activity, and medical history were collected by trained health workers using laptop-based questionnaires during the face-to-face interview. In addition, trained staff measured height and weight, waist and hip circumference, and blood pressure using standard instruments and protocols at baseline. Body mass index (BMI) was calculated by dividing weight (kg) by the square of height (m). The physical activity questionnaire collected information about the type and duration of activities related to work, transportation, housework, and leisure-time exercise during the past year. Metabolic equivalent task-hours per day (MET-h/d) were calculated to quantify the total amount of physical activity.

### Measurement of oral hygiene

Self-reported measure of frequency of toothbrushing was used as a proxy indicator of oral hygiene.^8,9^ An interviewer-administered questionnaire was used to collect information on gum bleeding. In the baseline questionnaire we asked the participants “How often do your gums bleed when you brush your teeth?”: “Occasionally, rarely or never”, “Sometimes”, “Always”, and “Brush teeth rarely or never”. Eight years after the baseline survey, a re-survey among 25,034 participants (4.9%) randomly selected from all surviving CKB participants was undertaken, collecting same information (including oral hygiene) as in the baseline survey. For toothbrushing question, the agreement rate between the two surveys was 91.2% (Kappa=0.352), while for gum bleeding, it was 59.8% (Kappa=0.233), indicating good consistency in reporting.

### Morbidity and mortality follow-up

We identified cause-specific morbidity and mortality during the follow-up using linkage with local disease and death registries, with the national health insurance system, and by active follow-up. All ten study areas are covered by the Chinese Disease Surveillance Points system, which provides cause-specific mortality data. The primary outcomes examined in the present study were first major vascular events (MVEs, composite of stroke, myocardial infarction [I21-I23] and cardiovascular death), and major coronary events (MCEs) (fatal ischemic heart disease (IHD) [ICD-10: I20-I25] or non-fatal myocardial infarction (MI) [I21-I23]), ischemic stroke (IS) (I63), intracerebral hemorrhage (ICH) (I61), cardiovascular death (I00-I99). Data on non-fatal disease outcomes (cardiovascular events, type 2 diabetes, cancer, chronic obstructive pulmonary disease (COPD), chronic kidney disease (CKD), and liver cirrhosis) were identified from the established local chronic disease registries and from national health insurance system, which has almost universal coverage (approximately 99%) in all study regions. The underlying causes of death were coded by trained staff members who used International Statistical Classification of Diseases and Related Health Problems, Tenth Revision (ICD-10) and were blinded to the baseline information. Residential records were used to identify participants who moved out of the study areas and were lost to follow-up.

### Statistical methods

We excluded participants with no follow-up in the age range 30-79 years (n=1), and those with a history of vascular disease (n=23,129; stroke, or transient ischaemic attack) and cancer (n=2,578) at baseline, leaving 487,198 in the main analyses. For prospective analyses of each non-vascular disease, participants with a history of the relevant disease at baseline were excluded. Analyses were censored at first event of the outcome of interest, death and lost to follow-up, whichever came first.

We used Cox regression analysis to estimate adjusted hazard ratios (HRs) for the associations between oral hygiene and first occurrence of MVE, CKD, type 2 diabetes, cancer, liver cirrhosis, COPD and all cause or cause specific mortality. HRs are stratified by age (5 years) and study area, and adjusted for sex (male or female), income (<20,000 yuan/year, or ≥20,000 yuan/year), educational level (no formal school, primary school, middle school, high school, college, or university or higher), marital status (married, widowed, divorced or separated, or never married), smoking status (never smoker, occasional smoker, former smoker, or regular smoker), alcohol intake (non-drinker, occasional drinker, former drinker, or regular drinker), physical activity (METs, h/day), frequency of fruit intake, vegetable intake, and meat intake (daily, 4 to 6 days/wk, 1 to 3 days/wk, monthly, or rarely or never), waist circumference, hip circumference, and systolic blood pressure. In categorical analyses, participants were divided into four groups according to oral hygiene: “Occasionally, rarely or never gum bleed (as reference)”, “Sometimes gum bleed”, “Always gum bleed”, and “Brush teeth rarely or never”. HRs were calculated relative to the “Occasionally, rarely or never gum bleed” group, and plotted against oral hygiene in each of these groups; the 95% CIs for these HRs were estimated with the variance of the log risk.^24^ Effect modification analyses were restricted to MVE. Assessment will be made for effect modification by: age at risk (30-49, 50-59, 60-69, and 70-79 years), and sex. For the overall associations with MVE, our sensitivity analyses assessed the effect of excluding first three years of follow-up. Reporting of the study conforms to broad EQUATOR guidelines.^25^

## Results

Among 487,198 adults included, the mean age at baseline was 51.5 years (SD 10.6) and 41% were men, and 43% resided in urban areas (Table 1). Overall, 9.3% participants reported rarely or never brushing teeth at baseline, with the proportion higher in men than women (11.4% vs 7.8%) but similar in rural and urban areas (9.5% vs 9.1%). Individuals who rarely or never brushed teeth were more likely to be men, older, and to have poor healthy lifestyle (e.g. more smoking, but less vegetable and fruit intakes) than those who regularly brushed teeth.

**Figure 1:**
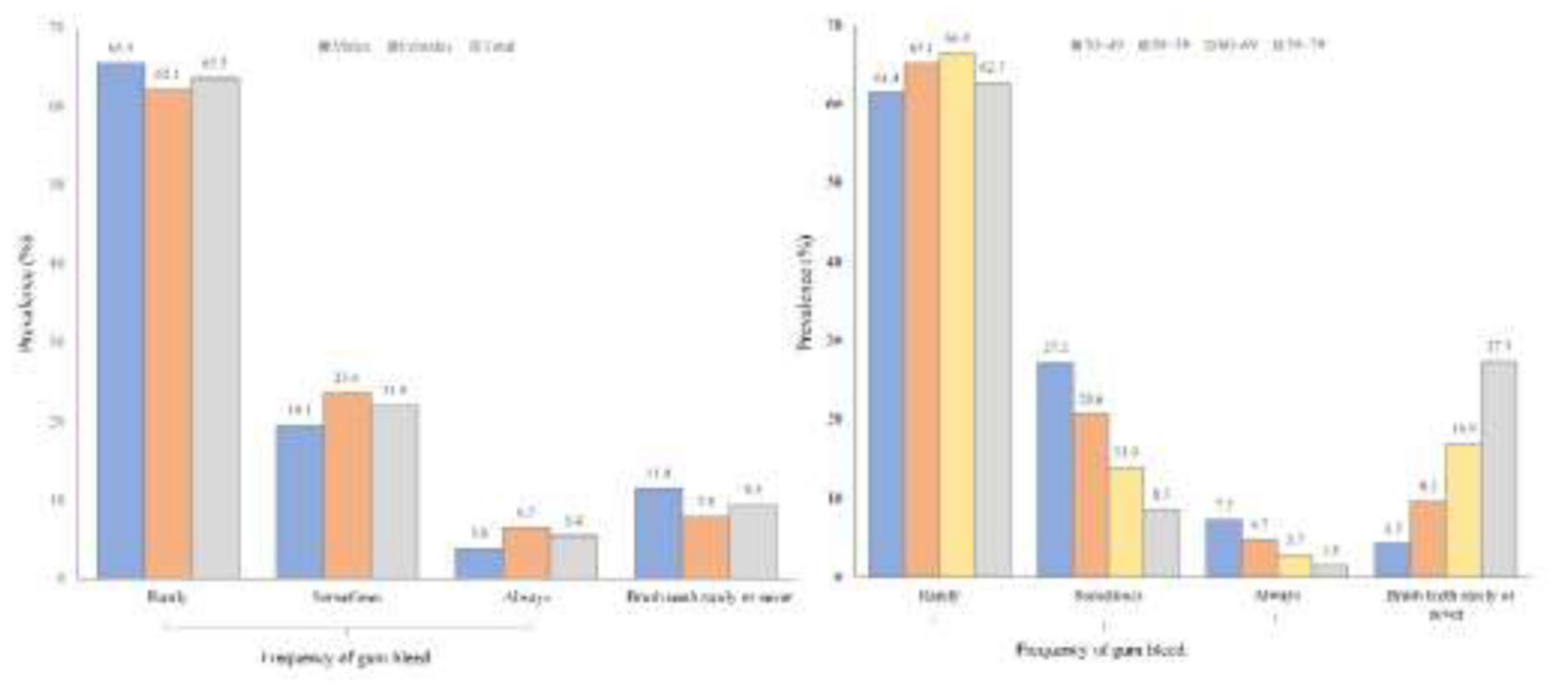
Age- and sex-specific prevalences of toothbrushing or gum bleeding among Chinese adults The prevalences of toothbrushing or gum bleeding among men and women or different age groups are shown.

Among those who brushed teeth regularly, the proportions who reported rarely, sometime and always having gum bleeding were 63.5%, 21.9% and 5.4%, respectively. The proportion of always having gum bleeding was higher in women than men (6.5% vs 3.8%). The percentage of participants who rarely or never brushed teeth was gradually increased with age whilst the converse was true for the percentage of gum bleeding (Figure 1).

**Figure 2:**
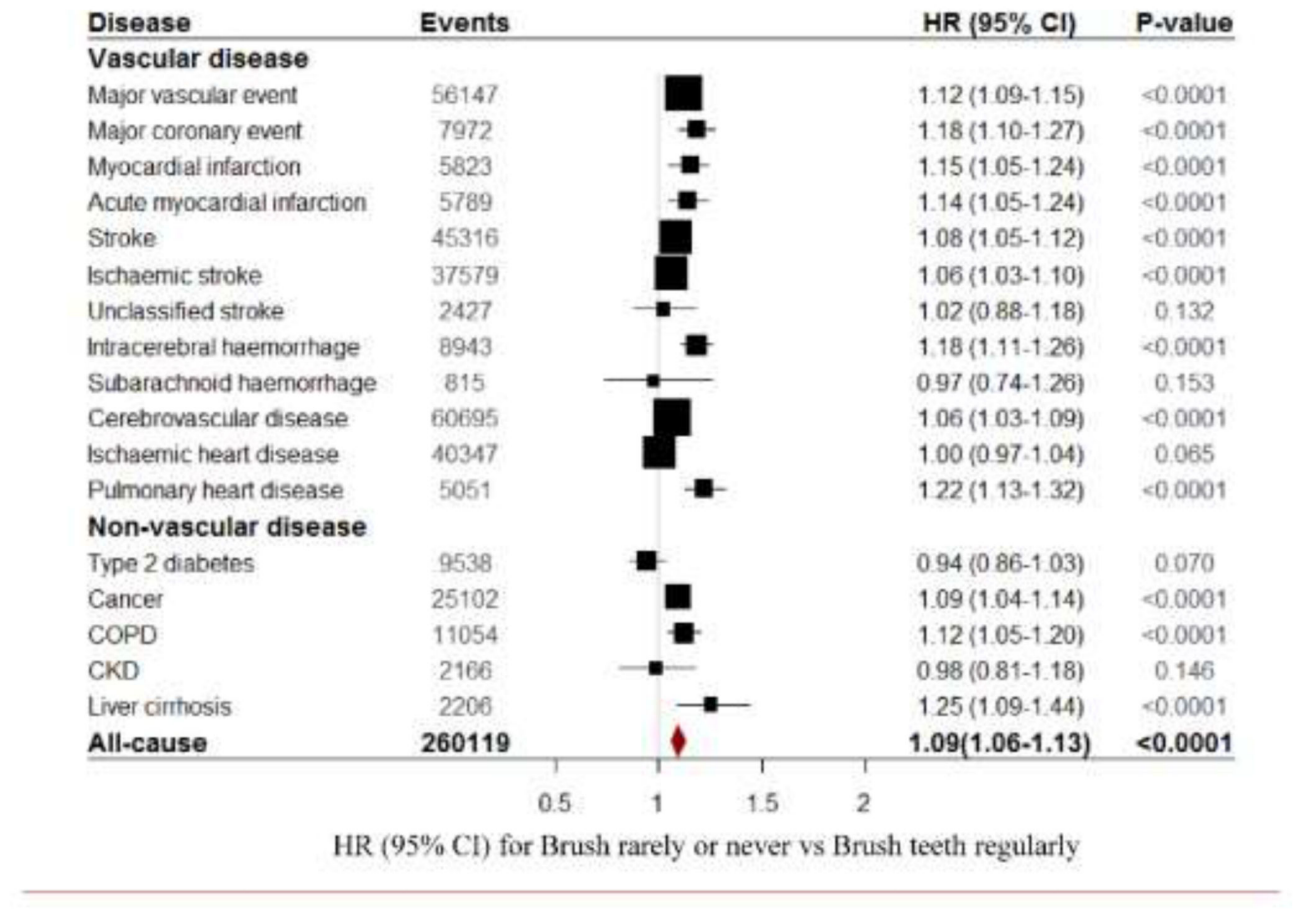
Major vascular disease and non-vascular disease by frequency of toothbrushing. HRs for frequently brush teeth vs rarely or never brush teeth at ages 30-79 years, adjusted for age, sex, area, education, smoking, alcohol intake, and BMI. The area of the square is inversely proportional to the variance of the log HR, which also determines the 95 %CI.

**Figure 3:**
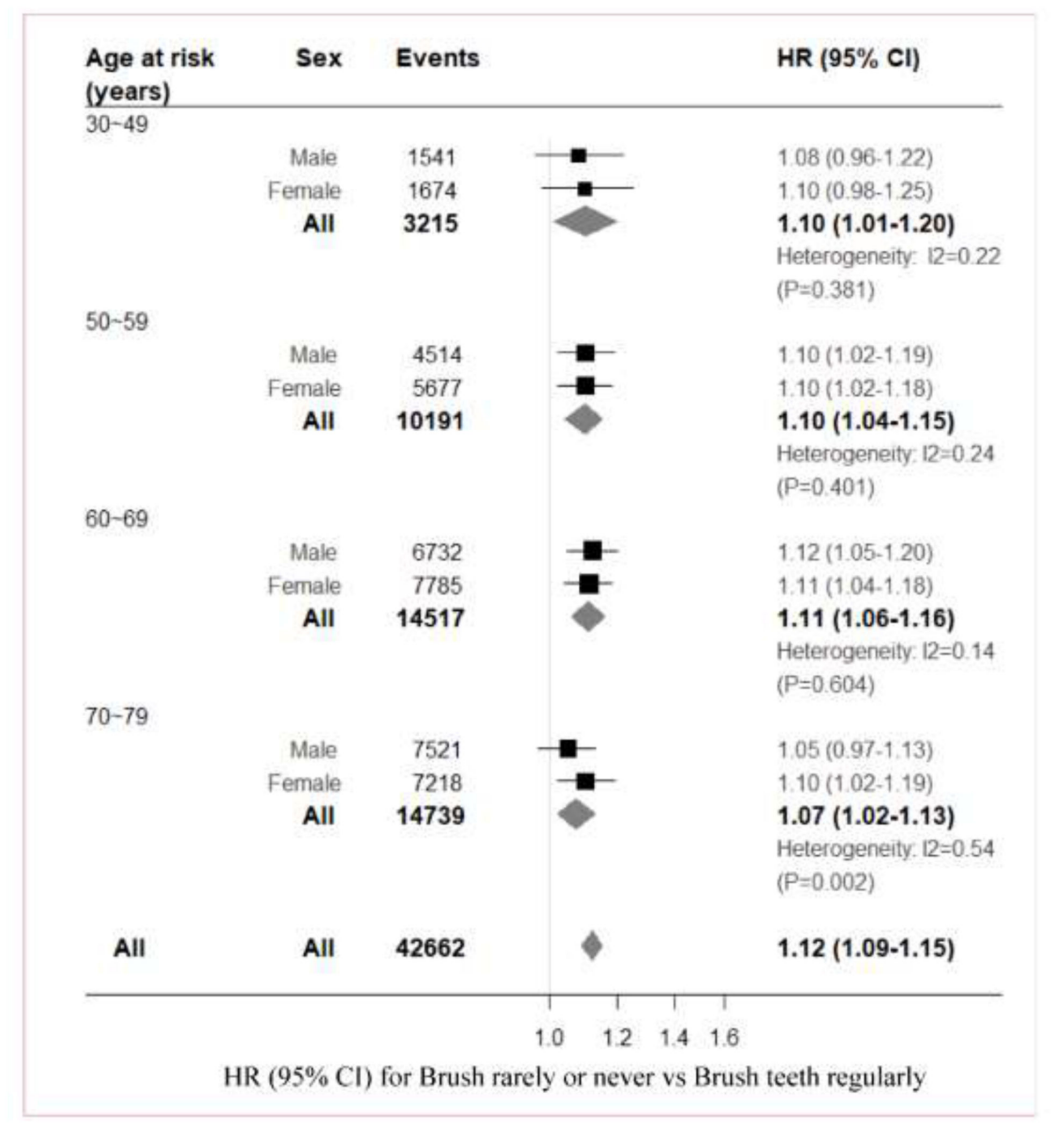
Age-specific incidence of major vascular events by frequency of toothbrushing HRs are stratified by study area and adjusted for age, sex, education, income, smoking, alcohol consumption, physical activity, fruit and vegetable intakes, meat intake, body-mass index, waist:hip ratio and systolic blood pressure. The area of each square is inversely proportional to the variance of the log HR. Corresponding 95% CIs are plotted as lines. HR=hazard ratio.

During a median 9.6 years of follow-up, 56,147 incident MVE occurred, including 7,972 MCE, 40,347 IHD, 37,579 IS, 45,316 total stroke, 8,943 ICH, 815 subarachnoid haemorrhage, and 2,427 of other (or unspecified) stroke (eTable 1). Overall, compared with those who brushed teeth regularly, those never or rarely brushed teeth had higher risk of MVE (HR, 1.12 [95% CI, 1.09, 1.15]), with similar risk estimates for stroke (1.08, 1.05-1.12), intracerebral haemorrhage (1.18, 1.11-1.26), and pulmonary heart disease (1.22, 1.13-1.32), whilst, no significant results were observed for subarachnoid haemorrhage (0.97, 0.74-1.26) and IHD (1.00, 0.97-1.04) (Figure 2). For each decade of age at risk, poor oral hygiene (rarely or never brushed teeth) was associated with higher risk of MVE (Figure 3). Females who rarely or never brushed teeth had similar risk for MVE (HR, 1.12 [95% CI, 1.08, 1.17]) with those for males (HR, 1.09 [95% CI, 1.05, 1.13]) (eTable 2). In addition, during follow-up, 9,538 incident type 2 diabetes, 25,102 cancer, 11,054 COPD, 2,166 CKD, 2,206 liver cirrhosis and 37,870 all cause deaths occurred (eTable 1). Those who did not brush teeth also had increased risk of cancer (1.09, 1.04-1.14), COPD (1.12, 1.05-1.20), liver cirrhosis (1.25, 1.09-1.44), and all cause death (1.25, 1.21-1.28), but not type 2 diabetes (0.94, 0.86-1.03) and CKD (0.98, 0.81-1.18) (Figure 2 & eFigure 1).

**Figure 4:**
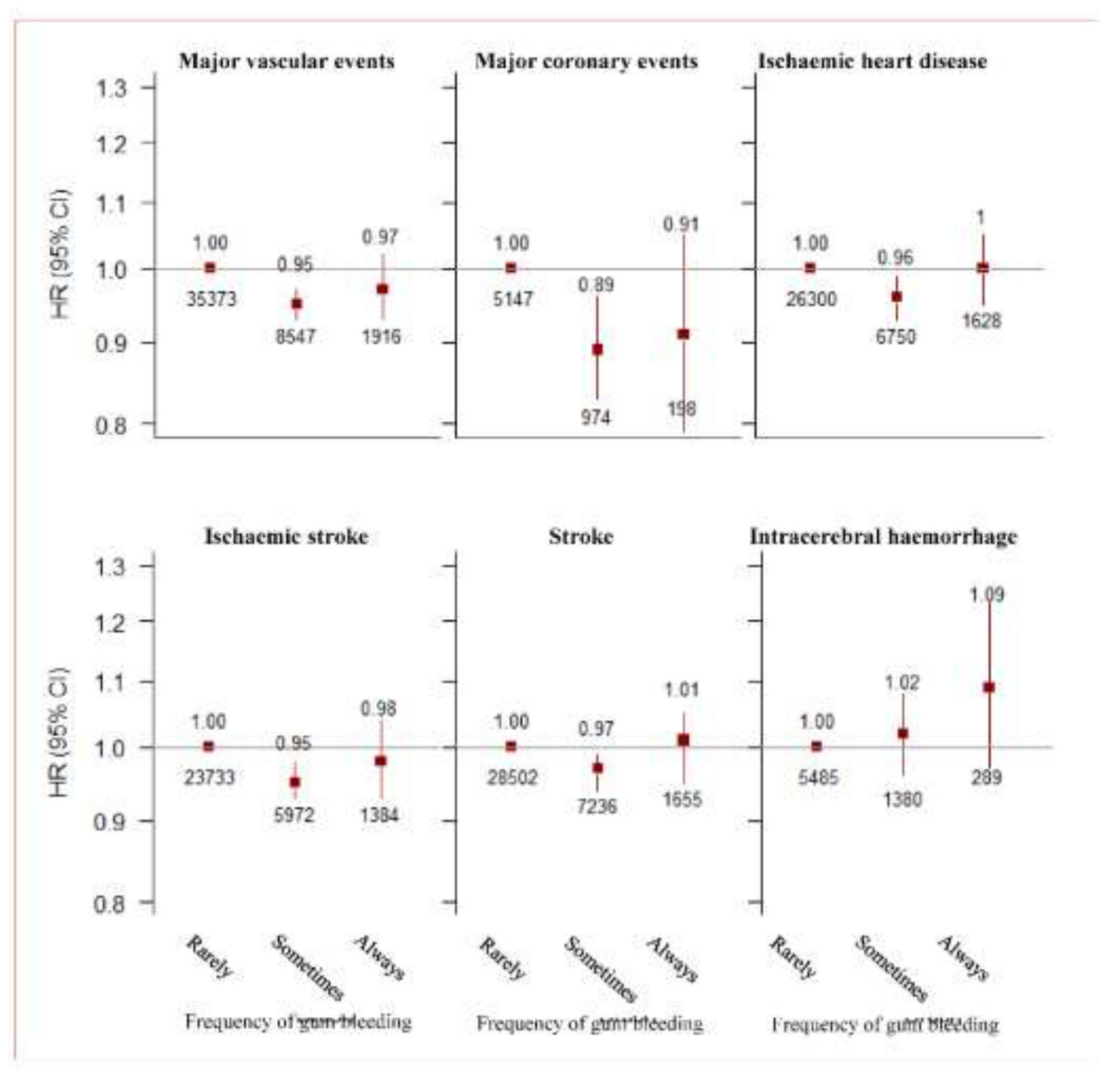
Observational association of frequency of gum bleeding with vascular diseases. Results are stratified by study area, and adjusted for age, sex, income, educational level, marital status, smoking status, alcohol intake, physical activity, frequency of fruit intake, vegetable intake, self-reported general health status, body-mass index, waist:hip ratio and systolic blood pressure.

**Figure 5:**
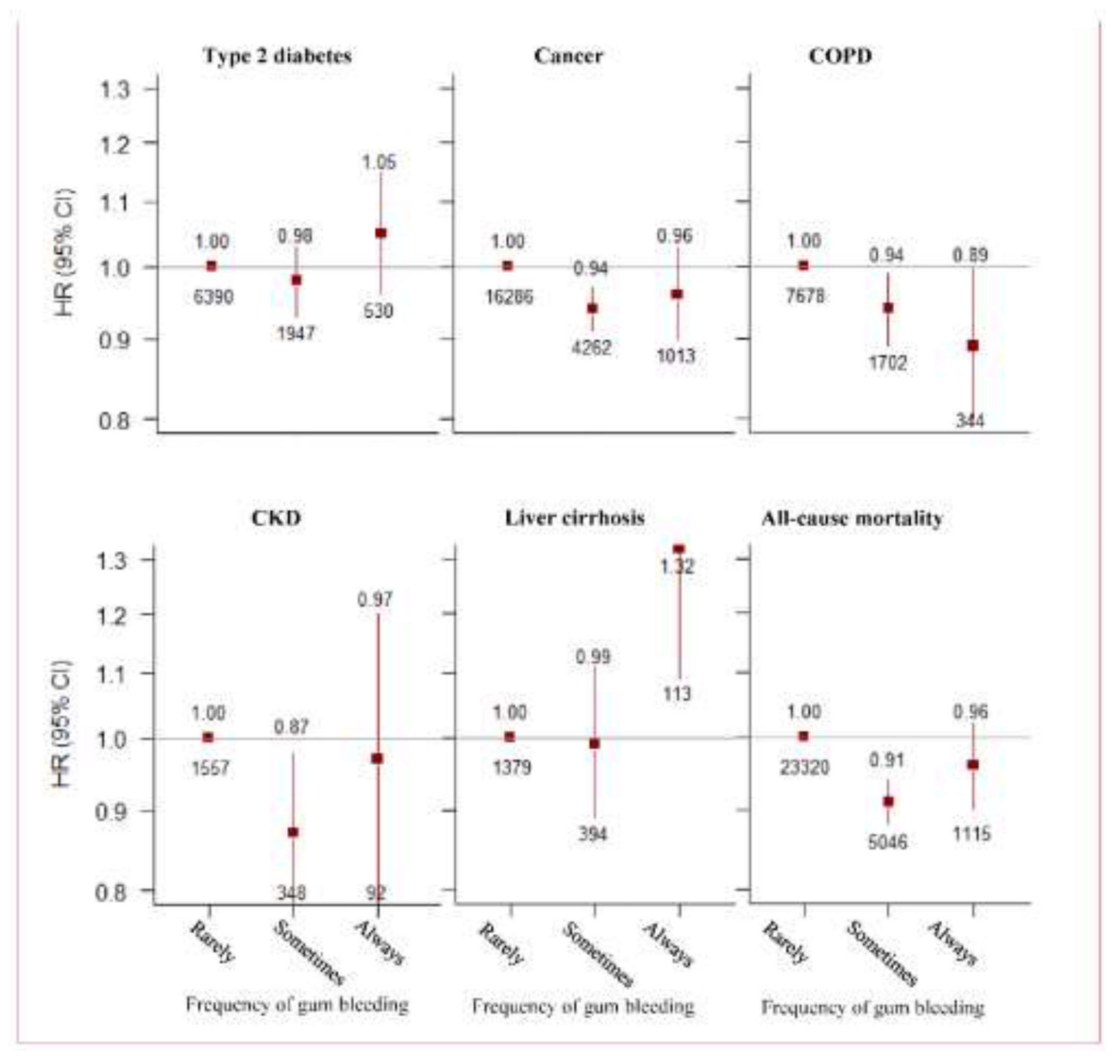
Observational association of frequency of gum bleeding with non-vascular diseases. Results are stratified by study area, and adjusted for age, sex, income, educational level, marital status, smoking status, alcohol intake, physical activity, frequency of fruit intake, vegetable intake, self-reported general health status, body-mass index, waist:hip ratio and systolic blood pressure.

Among those who brushed teeth regularly, those with more frequent (always) gum bleeding had no association with MVE (0.97, 0.93-1.02), whilst, those with less frequent (sometimes) gum bleeding had lower risk of MVE (0.95, 0.93-0.97), compared with those who never or rarely had gum bleeding. Similar risk estimates for MCE, IHD, IS, stroke, and non-vascular diseases such as cancer, and CKD (Figure 4 & 5). However, we did not observe significant association of frequency of gum bleeding with intracerebral haemorrhage, type 2 diabetes, liver cirrhosis (Figure 4 & 5).

For cause specific mortality, there were 14,182 deaths from MVE, 122 deaths from type 2 diabetes, 12,627 deaths from cancer, 1,281 deaths from COPD, 246 deaths from CKD and 304 deaths from liver cirrhosis during follow-up (eTable 3). Compared with frequent toothbrushing, the multivariable adjusted HRs for less frequent toothbrushing (rarely or never) were (HR, 1.28 [95% CI, 1.21, 1.34]) for major vascular deaths, (HR, 1.23 [95% CI, 1.13, 1.33]) for major coronary deaths, (HR, 1.23 [95% CI, 1.13, 1.33]) for IHD caused deaths, (HR, 1.13 [95% CI, 1.06, 1.19]) for cancer caused deaths, (HR, 1.42 [95% CI, 1.23, 1.64]) for COPD caused deaths, and (HR, 1.49 [95% CI, 1.05, 1.28]) for liver cirrhosis caused deaths (eFigure 1). However, toothbrushing behavior was not associated with death from type 2 diabetes and CKD.

In sensitivity analyses, the associations were unchanged by further adjustment for other potential confounders (including physical activity, frequency of fruit, vegetable and meat consumptions, medical history, BMI and WHR) (eTable 4); and excluding the first 3 years of follow-up (to assess for reverse causality) (eTable 5).

## Discussion

To our knowledge, our study is the largest prospective cohort study investigating the association of toothbrushing behavior with long-term follow-up of a wide range of clinically validated disease outcomes (vascular and non-vascular diseases) to date. In this large-scale prospective study among Chinese adults, we found that less frequent toothbrushing (never or rarely) was associated with higher risk for vascular events, and non-vascular disease such as cancer, COPD, liver cirrhosis, and all cause deaths. Our results also suggested that there was no association between those with more frequent (always) gum bleeding and the risk of major vascular and non-vascular diseases, but those with less frequent (sometimes) gum bleeding may have lower risks of vascular diseases, cancer, COPD, and CKD, compared with those who rarely had gum bleeding.

Many prospective studies in the Western populations have suggested the association between oral hygiene and CVD risk. In general, they consistently showed that poor oral hygiene was associated with higher risk of CVD.^8-11^ A national population based survey among 11 869 men and women (mean age 50) in Scotland indicated that participants who reported poor oral hygiene (never/rarely brushed their teeth) had an increased risk of CVD incidence (HR 95%CI: 1.7, 1.3 to 2.3), but not mortality from CVD (1.5, 0.9 to 2.6).^8^ These relative risk estimates for cardiovascular diseases was greater than that shown in the present study.^8^ However, the Scottish study included much smaller number of cases (n=555) than did the present study and were not able to examine reliably the association with risk of specific CVD types. It is worth noting that no prospective cohort study in Asian population has focused on the associations between oral hygiene and cardiovascular diseases. A cross-sectional Japanese study among 85 866 individuals showed that a lower frequency of toothbrushing was associated with high prevalences of cardiovascular risk factors such as hypertension, diabetes mellitus, dyslipidaemia.^9^ Similarly in Japan, a cohort study in 13,070 subjects reported associations of toothbrushing with cardiovascular risk factors and found that toothbrushing practices 'not after every meal' was a significant risk factor for developing diabetes in male and developing dyslipidemia in female compared with toothbrushing practices 'after every meal.^26^ However, another 5-year retrospective cohort study in Japanese reported that positive associations of toothbrushing with such risk factors were observed without sex difference.^27^ Therefore, whether toothbrushing has gender specific association with cardiovascular diseases remains unclear. Our study based on national wide large-scale prospective study demonstrated that the HRs for major vascular events were similar in female and in male.

However, none of the previous studies included sufficient numbers of well characterised vascular types such as stroke, intracerebral haemorrhage, IHD and pulmonary heart disease as in this study. In the present study, we specifically examined subtypes of vascular diseases and demonstrated that low frequency of toothbrushing was associated with higher risk for some (eg, stroke, intracerebral haemorrhage, and pulmonary heart disease), but not other CVD types (eg, subarachnoid haemorrhage and IHD). The reasons for the discrepant findings by CVD types were not clear and require further investigation.

Systemic inflammation may at least partly explain the underlying mechanism that links oral health with vascular disease.^6,10^ Inflammation plays an important role in the pathogenesis of atherosclerosis.^8^ Poor oral hygiene adds to the inflammatory burden such as increased concentrations of both C reactive protein and fibrinogen,^8^ and leads to increased cardiovascular risk. Therefore, poor oral hygiene itself may cause vascular disease. In support of this causal relationship, previous study demonstrated that periodontal treatment lowers C-reactive protein and low-density lipoproteins and improves endothelial function.^28,29^ Given the observed association between dental disease and vascular disease,^6-9^ future experimental studies are required to investigate whether the such observations is in fact causal or merely a risk marker.

Few previous studies have examined the associations of oral hygiene with risks of non-vascular diseases. In present study, we demonstrated that less frequent toothbrushing was associated with higher risk of cancer incidence and mortality. These results are in line with previous findings linking poor oral hygiene with higher risk of head and neck cancer,^30^ and esophageal carcinoma.^31^.^32^ Moreover, poor oral hygiene has also been associated with risk of chronic respiratory diseases in both population and clinical settings.^21,33^ In a study of 392 Chinese COPD patients, low toothbrushing times were associated with COPD exacerbations^.21^ In the present study, we found that both toothbrushing and gum bleeding were associated with higher risk of COPD. Our findings were supported by previous evidence that gum bleeding always/often was associated with higher risks of asthma symptoms and self-reported COPD.^34^. In addition, bacterial infections are frequent complications in patients with liver cirrhosis. Dental foci are a potential source of infection. Our study for the first time provided evidence that low frequent toothbrushing was associated with higher risk of liver cirrhosis. It remains largely unknown if liver cirrhosis itself is a predisposing factor for dental and periodontal diseases, or a consequence of poor oral hygiene. Prospective cohort studies are needed to confirm our findings.

A few studies have reported positive associations of toothbrushing and risk of DM, but these were mainly based on cross-sectional studies.^9,26,27^ However, we did not observe associations between toothbrushing and risk of type 2 diabetes and CKD in the present study. A single-centre, cross-sectional study among 85 866 individuals,^9^ a routine health examination study in 54,551 residents of Chiba City,^27^ and a 5-year retrospective cohort study in 13,070 Japanese adults consistently reported that lower frequency of teeth brushing is related to higher prevalence of diabetes mellitus. Our large scale prospective study included a ten times greater number of cases than did the Japanese studies and did not confirmed such associations in Chinese adults. The cross-sectional and retrospective study design used in Japanese adults at least partly may explain this discrepancy. In our study, we carefully controlled established confounding factors and excluded the first three years incident cases to overcome reverse causality, suggesting the robustness of the present findings.

Periodontal disease, which is linked to gum bleeding in response to plaque accumulation, could play a role in the initiation or progression of vascular disease.^12^ Few epidemiological studies have examined the associations between self-reported gum bleeding and chronic disease.^13,35^ Several case-control and cohort studies reported inflammatory periodontal disease was associated with higher risk of vascular disease,^36,37^ but the Physicians’ Health Study I in 22,071 U.S. male physicians reported that self-reports of presence or absence of periodontal disease was not associated with nonfatal myocardial infarction, nonfatal stroke and cardiovascular death. After adjustment for other cardiovascular risk factors, the associations were all further attenuated and nonsignificant.^13^ Similarly, we found that self-reported frequent gum bleeding was not associated with vascular and non-vascular diseases in the large scale prospective study among Chinese adults. Such conflicting evidence suggests the currently available evidence was insufficient to establish a cause-and-effect relationship between periodontal disease and vascular disease.^12-15^ Unexpectedly, we found a lower risk of vascular disease among those with less frequent (sometimes) gum bleeding. Thus, lower risk might be due to good oral hygiene among those who regularly brushed teeth.

Several strengths of the present study merit consideration, including a large sample size, a prospective cohort design, and careful control for established and potential risk factors for death. The large number of disease events and high-quality toothbrushing behaviour measurements by well trained technicians allowed us to gain sufficient power for conclusive estimation of associations. Further, we included participants at random and consecutively from the general population to minimized the potential for selection bias. Importantly, the main findings support health benefits of toothbrushing behavior in China and indicate that regular toothbrushing can have a substantial effect on cardiovascular risk.

However, the results should be interpreted with caution. First, oral hygiene and risks of chronic diseases may be have common risk factors. For example, previous study has suggested the impact of poor control for smoking on the association between oral health and systemic diseases.^38^ We could not exclude the possibility that the low HRs in our study might result from residual confounding due to smoking. In addition, residual confounding by unknown or unmeasured factors such as drug abuse was still possible, although we carefully adjusted for established risk factors for each outcome. It has been proven that antihypertensive drugs, such as calcium antagonist and amlodipine, may cause side events including gingival hyperplasia which may be attributed to bleeding or inflammation.^39,40^ Future study should take the effect of drugs induced bleeding and gum disease into consideration. Second, reverse causality might explain our findings to some extent because people with chronic disease might change their lifestyle, for example, frequently brushing tooth. However, the results remained largely unchanged after excluding participants with prior cancer and vascular disease at baseline and excluding incidence during the first three years of follow-up. Third, we could not rule out the possibility of misclassification of toothbrushing frequency. Such misclassification might produce bias in estimating hazard ratio. The questionnaire on toothbrushing used in our study has not yet been validated directly. In addition, measurement errors may be non-differential in a prospective study design. Therefore, the measure of association is more likely to be biased toward the null. Fourth, we only focused on major vascular events and major coronary events while did not test the association for other vascular diseases such as atrial fibrillation and heart failure in the present study, which have been demonstrated to be associated with improved oral hygiene care.^41^ Fifth, we haven’t assessed the associations for the number of teeth, the periodontal status and dental plaque without related information in CKB, which are important measurements for oral hygiene. Further studies are needed to examine the associations between the number of teeth, the periodontal status or dental plaque and risks of chronic diseases to complement our findings. Finally, we did not consider the change of toothbrushing behavior over time. The toothbrushing frequency reported for a short period may not necessarily reflect the long-term oral hygiene.

In summary, this large-scale analysis provides reliable evidence that less frequency of toothbrushing was associated with higher risks of major vascular disease and all-cause mortality. Given the strength of the associations between toothbrushing and vascular diseases, promotion of toothbrushing would be beneficial for prevention of vascular disease. However, the causal nature of such association is still needed to be determined.

## Supplementary Material

supplementary

## Figures and Tables

**Table 1:** Characteristics of the study population

Characteristics	Frequency of toothbrushing			Frequency of gum bleeding			
	Rarely or never	Sometimes or always	P value	Rarely	Sometimes	Always	P value
No. of participants	45299	441899		309229	106501	26169	
Demographic factors							
Age, y	58.2	50.3	<0.001	51.4	48.0	47.0	<0.001
Male, No. (%)	50.3	39.9	<0.001	42.2	36.3	28.7	<0.001
Urban region, No. (%)	9.1	46.9	<0.001	46.2	46.0	52.1	<0.001
Socioeconomic factors, No. (%)							
Household income >20,000 yuan/year, No. (%)	35.2	43.3	<0.001	45.6	45.0	43.6	<0.001
High school education and above, No. (%)	38.8	50.2	<0.001	51.8	51.1	50.1	<0.001
Married, No. (%)	86.5	91.4	<0.001	91.6	91.7	91.7	0.466
Lifestyle factors							
Physical activity, MET-h/d	21.9	21.5	<0.001	21.8	22.0	22.1	<0.001
Regular drinker, No. (%)	12.6	15.4	<0.001	15.6	14.8	15.5	<0.001
Regular smoker, No. (%)	29.6	26.4	<0.001	27.6	22.4	19.3	<0.001
Meat intake (day/week)	3.3	3.7	<0.001	3.9	3.8	3.9	0.004
Vegetable intake (day/week)	6.7	6.8	<0.001	6.9	6.8	6.8	<0.001
Fruit intake (day/week)	2.0	2.4	<0.001	2.6	2.4	2.4	<0.001
Self-reported conditions at baseline, No. (%)							
Diabetes	4.5	5.5	<0.001	5.3	5.3	5.7	0.062
Hypertension	30.7	34.1	<0.001	32.5	32.5	31.8	0.076
Poor health	11.9	9.0	<0.001	8.4	9.1	12.5	<0.001
Physical measurements, mean (SD)							
BMI, kg/m^2^	22.8	23.7	<0.001	23.7	23.6	23.5	<0.001
Waist hip ratio	0.9	0.9	<0.001	0.9	0.9	0.9	<0.001
SBP, mmHg	130.1	130.7	<0.001	129.9	129.9	129.3	0.001
DBP, mmHg	76.6	77.8	<0.001	77.6	77.6	77.4	0.068
Family medical history, No. (%)							
Diabetes	6.1	6.9	<0.001	6.9	7.3	7.7	<0.001
Cancer	15.7	16.7	<0.001	16.0	16.4	17.6	<0.001
Heart attack	2.7	3.2	<0.001	3.2	3.3	3.5	<0.001
Stroke	15.8	17.9	<0.001	17.0	17.6	18.2	<0.001
Season of recruitment (spring),							
No. (%)	31.1	30.0	<0.001	29.9	30.0	31.0	0.003

BMI, body mass index; SBP, systolic blood pressure; DBP, diastolic blood pressure; MET-h/d, metabolic equivalents of task per hours per day. Data are presented as mean ± SD for continuous variables and No. (%) for categorical variables, respectively. All variables are adjusted for age, sex and region when appropriate. All exposures were associated with toothbrushing behavior, with P<0.001.
