# Supplementary material for: Associations of toothbrushing behavior with risks of vascular and non-vascular diseases in Chinese adults
Source: Eur J Clin Invest. Author manuscript; Available in PMC 2024 Dec 5. (PMC7616900; doi:10.1111/eci.13634)
Supplement: supplementary [file EMS135885-supplement-supplementary.docx]

**eFigure 1. Major vascular disease and nonvascular disease death by toothbrushing.**


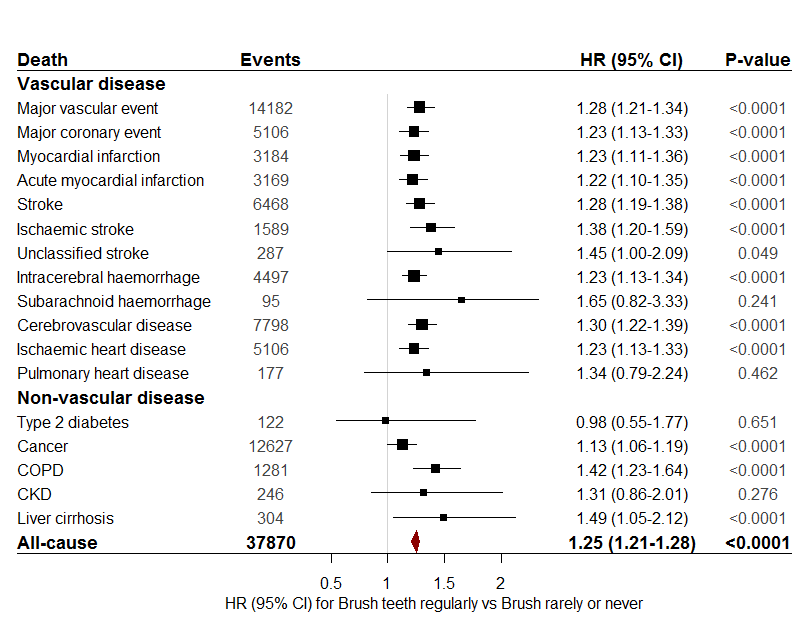


HRs for frequently brush teeth vs rarely or never brush teeth at ages 30-79 years, adjusted for age, sex, area, education, smoking, alcohol intake, and BMI. The area of the square is inversely proportional to the variance of the log HR, which also determines the 95 %CI.

eTable 1. Adjusted hazard ratios for vascular and non-vascular disease by categories of toothbrushing behaviors.

| **Disease** | **All participants** | | **Gum bleed** | | | | | | | | | | | **Brush teeth rarely or never** | | |
| --- | --- | --- | --- | --- | --- | --- | --- | --- | --- | --- | --- | --- | --- | --- | --- | --- |
|  |  |  | **Rarely** | | | **Sometimes** | | | | **Always** | | | |  |  |  |
| **No. of participants** | 441899 | |  | | |  | | | |  | | | | 45299 | | |
|  | **No. of person years** | **No. of all events** | **No. of person years** | **No. of events** | **Reference** | **No. of person years** | **No. of events** | **HR (95% CI)** | **p** | **No. of person years** | **No. of events** | **HR (95% CI)** | **p** | **No. of person years** | **No. of events** | **HR (95% CI)** |
| **Vascular disease** |  |  |  |  |  |  |  |  |  |  |  |  |  |  |  |  |
| Major vascular event | 4291067 | 45836 | 2974358 | 35373 | 1.00 | 1054960 | 8547 | 0.950(0.927-0.974) | <0.001 | 261749 | 1916 | 0.976(0.932-1.023) | 0.311 | 403893 | 10311 | 1.12 (1.09-1.15) |
| Major coronary event | 4418078 | 6319 | 3070862 | 5147 | 1.00 | 1079807 | 974 | 0.897(0.836-0.963) | 0.003 | 267408 | 198 | 0.919(0.796-1.061) | 0.250 | 428495 | 1653 | 1.18 (1.10-1.27) |
| Myocardial infarction | 4418078 | 4613 | 3070862 | 3713 | 1.00 | 1079807 | 753 | 0.909(0.839-0.986) | 0.021 | 267408 | 147 | 0.913(0.772-1.079) | 0.285 | 428495 | 1210 | 1.15 (1.05-1.24) |
| Stroke | 4300083 | 37393 | 2981534 | 28502 | 1.00 | 1056484 | 7236 | 0.967(0.942-0.993) | 0.015 | 262065 | 1655 | 1.003(0.953-1.054) | 0.921 | 404971 | 7923 | 1.08 (1.05-1.12) |
| Ischaemic stroke | 4314774 | 31089 | 2992352 | 23733 | 1.00 | 1059637 | 5972 | 0.953(0.926-0.982) | 0.001 | 262786 | 1384 | 0.982(0.929-1.037) | 0.506 | 407296 | 6490 | 1.06 (1.03-1.10) |
| Intracerebral haemorrhage | 4412026 | 7154 | 3066875 | 5485 | 1.00 | 1078089 | 1380 | 1.024(0.962-1.088) | 0.459 | 267062 | 289 | 1.097(0.973-1.238) | 0.131 | 426907 | 1789 | 1.18 (1.11-1.26) |
| Subarachnoid haemorrhage | 4424351 | 719 | 3076154 | 531 | 1.00 | 1080648 | 152 | 1.034(0.859-1.245) | 0.723 | 267548 | 36 | 1.030(0.730-1.453) | 0.865 | 429333 | 96 | 0.97 (0.74-1.26) |
| Unclassified stroke | 4420889 | 2155 | 3073342 | 1675 | 1.00 | 1080157 | 371 | 0.876(0.781-0.983) | 0.025 | 267390 | 109 | 1.056(0.867-1.285) | 0.591 | 428833 | 272 | 1.02 (0.88-1.18) |
| Cerebrovascular disease | 4245882 | 51304 | 2940481 | 39095 | 1.00 | 1046057 | 9895 | 0.966(0.945-0.989) | 0.003 | 259344 | 2314 | 1.015(0.972-1.059) | 0.502 | 399295 | 9391 | 1.06 (1.03-1.09) |
| Acute myocardial infarction | 4418117 | 4594 | 3070893 | 3697 | 1.00 | 1079816 | 750 | 0.909(0.838-0.986) | 0.021 | 267408 | 147 | 0.916(0.775-1.083) | 0.306 | 428533 | 1195 | 1.14 (1.05-1.24) |
| Ischaemic heart disease | 4298685 | 34678 | 2981043 | 26300 | 1.00 | 1056159 | 6750 | 0.962(0.936-0.989) | 0.006 | 261484 | 1628 | 1.001(0.952-1.054) | 0.955 | 411991 | 5669 | 1.00 (0.97-1.04) |
| Pulmonary heart disease | 4419800 | 3848 | 3072587 | 3076 | 1.00 | 1079811 | 643 | 0.783(0.717-0.855) | <0.001 | 267402 | 129 | 0.687(0.574-0.821) | <0.001 | 428013 | 1203 | 1.22 (1.13-1.32) |
| **Non-vascular disease** |  |  |  |  |  |  |  |  |  |  |  |  |  |  |  |  |
| Type 2 diabetes | 4170027 | 8867 | 2891102 | 6390 | 1.00 | 1026018 | 1947 | 0.978(0.928-1.030) | 0.392 | 252907 | 530 | 1.054(0.963-1.154) | 0.251 | 404269 | 671 | 0.94 (0.86-1.03) |
| Cancer | 4374804 | 21561 | 3039501 | 16286 | 1.00 | 1070248 | 4262 | 0.936(0.905-0.969) | 0.000 | 265055 | 1013 | 0.960(0.900-1.024) | 0.213 | 423232 | 3541 | 1.09 (1.04-1.14) |
| COPD | 4107964 | 9724 | 2842097 | 7678 | 1.00 | 1014851 | 1702 | 0.934(0.885-0.986) | 0.014 | 251016 | 344 | 0.890(0.797-0.993) | 0.038 | 380817 | 1330 | 1.12 (1.05-1.20) |
| CKD | 4356581 | 1997 | 3032268 | 1557 | 1.00 | 1062673 | 348 | 0.875(0.777-0.986) | 0.029 | 261640 | 92 | 0.973(0.785-1.205) | 0.800 | 426040 | 169 | 0.98 (0.81-1.18) |
| Liver cirrhosis | 4368557 | 1886 | 3038787 | 1379 | 1.00 | 1066368 | 394 | 0.999(0.890-1.121) | 0.981 | 263403 | 113 | 1.343(1.104-1.633) | 0.003 | 425696 | 320 | 1.25 (1.09-1.44) |
| **All-cause** | 2599282 | 235460 | 1774654 | 167658 | 1.00 | 662595 | 54610 | 0.966(0.956-0.975) | <0.001 | 162033 | 13192 | 0.990(0.972-1.008) | 0.253 | 244550 | 24659 | 0.98 (0.96-0.99) |

Results are stratified by study area, age (years) and adjusted for sex (male or female), income (<20,000 yuan/year, or ≥20,000 yuan/year), educational level (primary school or lower, middle school or higher), marital status (married, others), smoking status (current regular smoker, not current regular smoker), alcohol intake (current regular drinker, not current regular drinker), physical activity (METs, h/day), frequency of fruit intake, frequency of vegetable intake, frequency of meat intake, self-reported general health status, prevalent hypertension and diabetes at baseline (presence or absence); family history of cancer, heart attack, stroke, or diabetes (presence or absence), BMI and WHR.

eTable 2. Adjusted hazard ratios for major vascular events by categories of toothbrushing behaviors.

| **Major vascular events** | **Baseline HR (95%CI)** | | | |  |
| --- | --- | --- | --- | --- | --- |
|  | **Gum bleed** | | | **Brush rarely or never** | **Phtest** |
|  | **Never or rarely** | **Sometimes** | **Always** |  |  |
| **Gender** |  |  |  |  |  |
| Males | 1.00 | 0.94 (0.90-0.97) | 0.94 (0.87-1.02) | 1.09 (1.05-1.13) | <0.001 |
| Females | 1.00 | 0.95 (0.92-0.98) | 0.99 (0.93-1.05) | 1.12 (1.08-1.17) | <0.001 |
| **Age group** |  |  |  |  |  |
| 30~49 | 1.00 | 0.99 (0.94-1.04) | 1.06 (0.97-1.15) | 1.10 (1.01-1.20) | <0.001 |
| 50~59 | 1.00 | 0.95 (0.91-0.99) | 1.00 (0.92-1.08) | 1.08 (1.02-1.14) | <0.001 |
| 60~69 | 1.00 | 0.95 (0.91-0.99) | 0.91 (0.83-1.00) | 1.10 (1.05-1.15) | <0.001 |
| 70~79 | 1.00 | 0.94 (0.87-1.01) | 0.92 (0.77-1.09) | 1.06 (1.01-1.12) | <0.001 |

Results are stratified by study area, age (years) and adjusted for sex (male or female), income (<20,000 yuan/year, or ≥20,000 yuan/year), educational level (primary school or lower, middle school or higher), marital status (married, others), smoking status (current regular smoker, not current regular smoker), alcohol intake (current regular drinker, not current regular drinker), physical activity (METs, h/day), frequency of fruit intake, frequency of vegetable intake, frequency of meat intake, self-reported general health status, prevalent hypertension and diabetes at baseline (presence or absence); family history of cancer, heart attack, stroke, or diabetes (presence or absence), BMI and WHR.

eTable 3. Adjusted hazard ratios for vascular and non-vascular mortality by categories of toothbrushing behaviors.

| **Deaths** | **All participants** | | **Gum bleed** | | | | | | | | | | | | **Brush teeth rarely or never** | | | |
| --- | --- | --- | --- | --- | --- | --- | --- | --- | --- | --- | --- | --- | --- | --- | --- | --- | --- | --- |
|  |  |  | **Rarely** | | | **Sometimes** | | | | **Always** | | | | |  |  |  |  |
| **No. of person years** | **441899** | | **309229** | | | **106501** | | | | **26169** | | | | | **45299** | | | |
|  | **No. of person years** | **No. of all events** | **No. of person years** | **No. of events** | **Reference** | **No. of person years** | **No. of events** | **HR (95% CI)** | **p** | **No. of person years** | **No. of events** | **HR (95% CI)** | **p** | **No. of person years** | | **No. of events** | **HR (95% CI)** |  |
| **Vascular death outcomes:** |  |  |  |  |  |  |  |  |  |  |  |  |  |  | |  |  |  |
| Major vascular event | 4426730 | 10532 | 3077873 | 8509 | 1 | 1081167 | 1686 | 0.928(0.879-0.979) | 0.007 | 267691 | 337 | 0.966(0.865-1.079) | 0.542 | 429557 | | 3650 | 1.28 (1.21-1.34) |  |
| Major coronary event | 4426730 | 3816 | 3077873 | 3137 | 1 | 1081167 | 568 | 0.883(0.805-0.968) | 0.008 | 267691 | 111 | 0.896(0.740-1.086) | 0.264 | 429557 | | 1290 | 1.23 (1.13-1.33) |  |
| Myocardial infarction | 4426730 | 2275 | 3077873 | 1838 | 1 | 1081167 | 372 | 0.901(0.803-1.012) | 0.080 | 267691 | 65 | 0.885(0.688-1.137) | 0.340 | 429557 | | 909 | 1.23 (1.11-1.36) |  |
| Stroke | 4426730 | 4847 | 3077873 | 3832 | 1 | 1081167 | 852 | 1.002(0.928-1.083) | 0.951 | 267691 | 163 | 1.038(0.885-1.218) | 0.646 | 429557 | | 903 | 1.22 (1.10-1.35) |  |
| Ischaemic stroke | 4426730 | 1102 | 3077873 | 910 | 1 | 1081167 | 160 | 0.842(0.708-1.001) | 0.051 | 267691 | 32 | 0.885(0.620-1.265) | 0.504 | 429557 | | 1621 | 1.28 (1.19-1.38) |  |
| Intracerebral haemorrhage | 4426730 | 3424 | 3077873 | 2662 | 1 | 1081167 | 640 | 1.065(0.973-1.166) | 0.173 | 267691 | 122 | 1.130(0.939-1.360) | 0.196 | 429557 | | 487 | 1.38 (1.20-1.59) |  |
| Subarachnoid haemorrhage | 4426730 | 80 | 3077873 | 61 | 1 | 1081167 | 17 | 1.117(0.641-1.947) | 0.697 | 267691 | 2 | 0.525(0.126-2.178) | 0.375 | 429557 | | 46 | 1.45 (1.00-2.09) |  |
| Unclassified stroke | 4426730 | 241 | 3077873 | 199 | 1 | 1081167 | 35 | 0.853(0.59-1.233) | 0.398 | 267691 | 7 | 0.777(0.362-1.669) | 0.518 | 429557 | | 1073 | 1.23 (1.13-1.34) |  |
| Cerebrovascular disease | 4426730 | 5642 | 3077873 | 4480 | 1 | 1081167 | 971 | 0.984(0.916-1.058) | 0.667 | 267691 | 191 | 1.032(0.891-1.195) | 0.677 | 429557 | | 15 | 1.65 (0.82-3.33) |  |
| Acute myocardial infarction | 4426730 | 2266 | 3077873 | 1830 | 1 | 1081167 | 371 | 0.902(0.803-1.014) | 0.084 | 267691 | 65 | 0.890(0.693-1.144) | 0.365 | 429557 | | 2156 | 1.30 (1.22-1.39) |  |
| Ischaemic heart disease | 4426730 | 3816 | 3077873 | 3137 | 1 | 1081167 | 568 | 0.883(0.805-0.968) | 0.008 | 267691 | 111 | 0.896(0.740-1.086) | 0.264 | 429557 | | 1290 | 1.23 (1.13-1.33) |  |
| Pulmonary heart disease | 4426730 | 143 | 3077873 | 125 | 1 | 1081167 | 18 | 0.830(0.497-1.388) | 0.478 | 267691 | 0 |  |  | 429557 | | 34 | 1.34 (0.79-2.24) |  |
| **Non-vascular death outcomes:** | |  |  |  |  |  |  |  |  |  |  |  |  |  | |  |  |  |
| Type 2 diabetes | 4203118 | 105 | 2914503 | 87 | 1 | 1033419 | 18 | 0.86(0.508-1.457) | 0.575 | 255197 | 0 |  |  | 406767 | | 17 | 0.98 (0.55-1.77) |  |
| Cancer | 4426730 | 10315 | 3077873 | 8022 | 1 | 1081167 | 1848 | 0.910(0.864-0.958) | <0.001 | 267691 | 445 | 1.024(0.930-1.129) | 0.626 | 429557 | | 2312 | 1.13 (1.06-1.19) |  |
| COPD | 4140898 | 888 | 2868013 | 744 | 1 | 1020682 | 128 | 0.856(0.703-1.041) | 0.120 | 252203 | 16 | 0.638(0.386-1.052) | 0.078 | 384269 | | 393 | 1.42 (1.23-1.64) |  |
| CKD | 4362960 | 207 | 3037217 | 172 | 1 | 1063788 | 28 | 0.856(0.566-1.293) | 0.459 | 261955 | 7 | 1.093(0.505-2.366) | 0.821 | 426455 | | 39 | 1.31 (0.86-2.01) |  |
| Liver cirrhosis | 4373230 | 249 | 3042233 | 169 | 1 | 1067294 | 67 | 1.415(1.057-1.895) | 0.020 | 263702 | 13 | 1.238(0.697-2.197) | 0.466 | 426319 | | 55 | 1.49 (1.05-2.12) |  |
| **All-cause** | 4426575 | 29481 | 3077743 | 23320 | 1 | 1081142 | 5046 | 0.911(0.883-0.940) | <0.001 | 267690 | 1115 | 0.967(0.910-1.028) | 0.285 | 429541 | | 8389 | 1.25 (1.21-1.28) |  |

Results are stratified by study area, age (years) and adjusted for sex (male or female), income (<20,000 yuan/year, or ≥20,000 yuan/year), educational level (primary school or lower, middle school or higher), marital status (married, others), smoking status (current regular smoker, not current regular smoker), alcohol intake (current regular drinker, not current regular drinker), physical activity (METs, h/day), frequency of fruit intake, frequency of vegetable intake, frequency of meat intake, self-reported general health status, prevalent hypertension and diabetes at baseline (presence or absence); family history of cancer, heart attack, stroke, or diabetes (presence or absence), BMI and WHR.

eTable 4. Effect of adjustment for confounders on the association of toothbrushing behaviors with major vascular events.

| **Toothbrushing** | **No. of participants** | **No. of person years** | **No. of events** | **HR (95% CI)** | | | | |
| --- | --- | --- | --- | --- | --- | --- | --- | --- |
|  |  |  |  | **Model1** | **Model2** | **Model3** | **Model4** | **Model5** |
| Rarely gum bleed | 309229 | 2974358 | 35373 | 1.00 | 1.00 | 1.00 | 1.00 | 1.00 |
| Sometimes gum bleed | 106501 | 1054960 | 8547 | 0.94 (0.92-0.97) | 0.95 (0.93-0.98) | 0.95 (0.92-0.97) | 0.95 (0.92-0.97) | 0.95 (0.93-0.97) |
| Always gum bleed | 26169 | 261749 | 1916 | 0.98 (0.94-1.03) | 1.00 (0.95-1.04) | 0.99 (0.95-1.04) | 0.97 (0.93-1.02) | 0.97 (0.93-1.02) |
| Brush teeth rarely or never | 45299 | 403893 | 10311 | 1.10 (1.07-1.13) | 1.10 (1.07-1.13) | 1.09 (1.06-1.12) | 1.10 (1.07-1.13) | 1.10 (1.07-1.13) |

Model1 adjusted for study area, age (years) and adjusted for sex (male or female) income (<20,000 yuan/year, or ≥20,000 yuan/year), educational level (primary school or lower, middle school or higher), marital status (married, others).

Model2 adjusted for variables in model1, smoking status (current regular smoker, not current regular smoker), alcohol intake (current regular drinker, not current regular drinker), physical activity (METs, h/day).

Model3 adjusted for variables in model2, frequency of fruit intake, frequency of vegetable intake, frequency of meat intake.

Model4 adjusted for variables in model3, self-reported general health status, prevalent hypertension and diabetes at baseline (presence or absence); family history of cancer, heart attack, stroke, or diabetes (presence or absence).

Model5 adjusted for variables in model4, BMI and WHR

eTable 5. Sensitivity analysis by excluding the first 3 years of follow-up.

| **Toothbrushing** | **No. of participants** | **No. of person years** | **No. of events** | **HR (95% CI)** |
| --- | --- | --- | --- | --- |
| Rarely gum bleed | 302891 | 2963643 | 29035 | 1.00 |
| Sometimes gum bleed | 105116 | 1052588 | 7162 | 0.96 (0.93-0.98) |
| Always gum bleed | 25872 | 261227 | 1619 | 0.98 (0.94-1.04) |
| Brush teeth rarely or never | 43105 | 400305 | 8117 | 1.08 (1.05-1.11) |

Results are stratified by study area, age (years) and adjusted for sex (male or female), income (<20,000 yuan/year, or ≥20,000 yuan/year), educational level (primary school or lower, middle school or higher), marital status (married, others), smoking status (current regular smoker, not current regular smoker), alcohol intake (current regular drinker, not current regular drinker), physical activity (METs, h/day), frequency of fruit intake, frequency of vegetable intake, frequency of meat intake, self-reported general health status, prevalent hypertension and diabetes at baseline (presence or absence); family history of cancer, heart attack, stroke, or diabetes (presence or absence), BMI and WHR.
